# Selection of the appropriate method for the assessment of insulin resistance

**DOI:** 10.1186/1471-2288-11-158

**Published:** 2011-11-23

**Authors:** Anwar Borai, Callum Livingstone, Ibrahim Kaddam, Gordon Ferns

**Affiliations:** 1 Department of Pathology, King Khalid National Guard Hospital, Jeddah, Saudi Arabia; 2 King Abdullah International Medical Research Center-Jeddah, Saudi Arabia; 3 Faculty of Health & Medical Sciences, University of Surrey, UK; 4 Clinical Biochemistry Department, Royal Surrey County Hospital, Guildford, UK; 5 Institute for Science and Technology in Medicine, University of Keele, UK

## Abstract

Insulin resistance is one of the major aggravating factors for metabolic syndrome. There are many methods available for estimation of insulin resistance which range from complex techniques down to simple indices. For all methods of assessing insulin resistance it is essential that their validity and reliability is established before using them as investigations. The reference techniques of hyperinsulinaemic euglycaemic clamp and its alternative the frequently sampled intravenous glucose tolerance test are the most reliable methods available for estimating insulin resistance. However, many simple methods, from which indices can be derived, have been assessed and validated *e.g*. homeostasis model assessment (HOMA), quantitative insulin sensitivity check index (QUICKI). Given the increasing number of simple indices of IR it may be difficult for clinicians and researchers to select the most appropriate index for their studies. This review therefore provides guidelines and advices which must be considered before proceeding with a study.

## Background

An index of insulin resistance (IR) can be defined as a quantitative measurement of the biological effect of endogenous or exogenous insulin in relation to the ambient blood glucose level. IR is considered to be an independent risk factor for the development of metabolic syndrome and diabetes. Predisposition to IR is multi-factorial, with strong genetic and environmental influences. Over recent years there has been widespread scientific interest in this topic as it has become apparent that IR develops early in the pathological process leading to diabetes. Many studies have shown that it may predate the onset of the diabetes by 10-20 years [1]. Quantitative assessment of IR may therefore be useful for detecting its presence and assessing its severity, particularly in subjects who have not as yet developed abnormal glucose tolerance or diabetes. Although the presence of IR can be inferred from clinical findings it is not currently common practice to quantitate it in clinical contexts. Its quantitation is largely confined to research studies.

In a previous literature review [2] we classified methods of assessing IR into three main categories *viz *dynamic tests, simple indices and biochemical markers. In the dynamic tests such as the hyperinsulinaemic euglycaemic clamp (HEC) and its alternative technique the frequently sampled intravenous glucose tolerance (FSIVGTT) blood samples are collected serially. These are considered reference techniques. Relatively simple, non-invasive alternatives to the clamp technique have been proposed, such as homeostasis model assessment (HOMA). In addition it has been observed that measurement of individual biochemical protein markers such as insulin-like growth factor binding protein-1 (IGFBP-1) can provide useful information about the status of IR [3-5].

Previously studies have discussed the available methods for assessing IR including their applications and limitations [6,7] while other suggested some general requirements for an ideal method for measuring IR [8,9].

Faced with the wide array of indices available, it may be difficult for investigators to decide which is the most suitable for a particular purpose. However, no previous single publication has discussed the various factors that need to be considered in method selection. In this review we have collated available information on these factors in an effort to guide investigators in their choice of method.

### General considerations for choosing the appropriate technique prior quantitating insulin resistance

**Figure 1:**
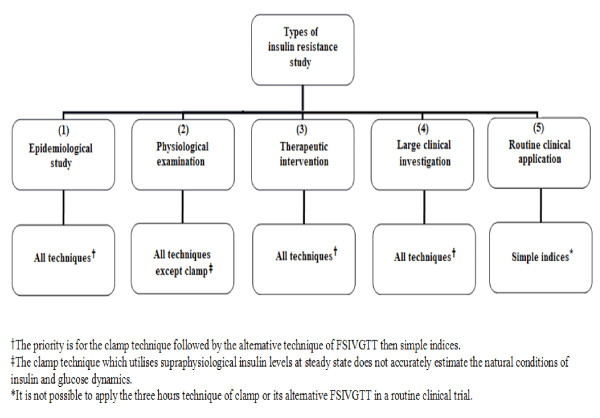
**Different types of insulin resistance studies**.

Investigators planning a study in which IR will be quantitated should in the first instance be clear about the type of study to be undertaken as this is the principle factor in determining the choice of method. The different types of study described in the literature and broad choice of method are summarised in Figure 1. The investigator should have prior knowledge of the available methods for assessing IR, their limitations and how to prepare subjects prior to investigation.

In making a decision, consideration should be given to the additional information required beyond the determination of IR. Some methods, particularly reference techniques, permit estimation of additional parameters of interest. Furthermore, some techniques provide an estimate of hepatic or peripheral IR or both. The investigator needs to consider whether the IR index is the primary endpoint of the study, such as would be the case in a study comparing IR between two populations, or of secondary interest. In selecting methodology there will be obvious logistical factors to consider. Below we consider in detail the different methods available for assessing IR and factors which investigators need to consider.

### Reference techniques

The HEC should be the test of first choice in studies where IR is of primary interest [10]. It is a steady-state technique which requires a constant insulin infusion. Clearly this is unphysiological thus the HEC is not appropriate when an estimation of insulin action and glucose dynamics under normal physiological conditions is required.

Owing to the complexity of the HEC, the FSIVGTT may be preferred as an alternative option and, as such, can be considered the 'silver' standard [11]. The main FSIVGTT techniques available are the standard (classical) technique and the more modern insulin-modified version. Simplified short sampling protocols have been described for ease of studying large numbers of subjects [12]. Unlike the HEC, the FSIVGTT relies on dynamic glucose and insulin data obtained before and following an intravenous glucose bolus. It therefore measures IR indirectly. All FSIVGTT techniques require minimal model analysis in order to derive the insulin sensitivity index, Si. This can only be done using software packages such as MINMOD. Many different software packages are available but do yield slightly different results. Investigators should endeavour to use the most modern version. In addition to determining Si, the differential equations used by the MINMOD program can be utilised to estimate many other parameters of interest *e.g*. glucose effectiveness (Sg), β-cell activity (β-cell), acute insulin response (AIR), disposition index (DI) and area under the curve (AUC). For investigators who are interested in these parameters the FSIVGTT will be the test of choice. However, it should be anticipated that for some subjects it may not possible to derive the various parameters from the available data. This is a common limitation of the technique encountered by authors, including ourselves, which should be taken into account when planning the number of study subjects to be recruited.

For investigators interested in assessing hepatic glucose production (HGP) and IR, stable isotopes of glucose can be used in combination with clamp or FSIVGTT techniques. For further details on this topic readers are directed to an appropriate reference [13,14]. Other dynamic techniques such as the insulin tolerance test (ITT) and continuous infusion of glucose with model assessment (CIGMA) have not been widely benchmarked and only a few groups have used them in their studies [15-17]. We will not therefore discuss them further here.

#### Number of subjects

**Figure 2:**
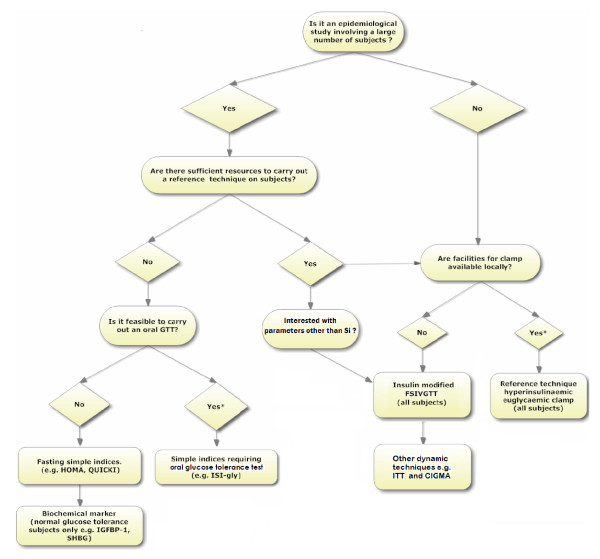
Protocol for selection the most appropriate technique for assessment of insulin resistance prior to commencement of a study.* This option is preferred. Key: FSIVGTT, frequently sampled intravenous glucose tolerance test; GTT, glucose tolerance test; HOMA, homeostasis model assessment; IGFBP-1, insulin-like growth factor binding protein-1; ISI-gly, insulin sensitivity index of glycaemia; ITT, insulin tolerance test; CIGM, continuous infusion of glucose with model assessment; QUICKI, quantitative insulin sensitivity check index; SHBG, sex hormone binding protein.

The number of subjects involved in a study is an important determinant of the feasibility of a given technique (Figure 2). It is well recognised that the reference techniques are generally unsuitable for epidemiological studies involving large numbers of subjects. However, there have been exceptions and, in principle, there is no reason why a reference technique should not be used in an epidemiological study provided sufficient resources are available. In the IR atherosclerosis study a large number of subjects (n = 1,625) was used to examine the association between Si derived from insulin-modified FSIVGTT and other cardiovascular risk factors [18]. In general however, reference techniques have been confined to studies with relatively low numbers of subjects, especially when it is a pilot study investigating a new treatment modality or where a novel method is being validated [5,19].

#### Glycaemic status of subjects

Knowledge of the subjects' glycaemic status influences the choice of method. Where subjects with diabetes are to be studied, the HEC or insulin-modified FSIVGTT should be used. The classical FSIVGTT relies solely on the endogenous insulin response which, in diabetic subjects, may be insufficient to permit determination of Si. In addition, short protocol FSIVGTTs are best avoided in subjects with diabetes owing to poorer correlation of Si with the gold standard in these subjects. Clearly the glycaemic status of study subjects should be known by the investigator beforehand.

#### Budget

Budgetary constraints will clearly impact on the choice of the method. Where sufficient funds are available, the HEC reference technique is first choice but if this is not possible then the FSIVGTT would be recommended. However, it should be noted these methods cost 20-30 times more than the simple indices. Both the HEC and FSIVGTT require a dedicated member of staff with the necessary expertise. In the authors' experience it is desirable, in addition, to have a second member of staff available with expertise in the technique during attendance of study subjects, a factor worth considering in planning. Clearly this must be taken into account in budgetary considerations as staffing is likely to be the most significant contributor to the cost of the research.

#### Materials and equipment

The availability of appropriate technical expertise and materials must be considered if a reference technique is to be used. Essentially these techniques should be undertaken in a fully equipped clinical investigation unit in the presence of a suitably trained member of staff (*e.g*. a nurse) able to manage any complications which may arise. A field study involving subjects who are unable to travel to the research centre would clearly preclude the use of a reference technique and demand use of simple technique on a single sample which could be processed and transported appropriately to the laboratory.

Experience with data analysis is another important factor to consider. If for example a software package such as MINMOD is to be used in conjunction with the FSIVGTT, for sophisticated data analysis, the operator should have the necessary skills for analysing the data and have access to support [20]. Both the HEC and FSIVGTT require cannulation of subjects, unlike the simple IR indices which only require straightforward venesection. The subjects' veins therefore need to be of sufficient quality if they are to be included and an operator must be present who possesses the necessary expertise.

#### Previous studies

Before utilising a reference technique in a study, it is advisable to share information with those with previous expertise in the technique so that potential difficulties and hazards, such as the risk of insulin-induced hypoglycaemia, can be identified. This will also enable the researcher to ascertain necessary details such as the protocol for use, possible application, pitfalls and limitations.

### Simple indices of insulin resistance

A simple index of IR can be defined as an index which does not require the intravenous administration of exogenous insulin or glucose. It can be estimated either from a fasting specimen alone or fasting specimen along with other blood samples taken following an oral glucose load. Simple indices, unlike dynamic techniques, do not demand steady state conditions. Owing to their ease of application and convenience to subjects, simple indices are the most commonly used tools for estimating IR. Therefore, it is important to be aware whether a simple index of IR can or cannot be utilised in a given study. Criteria for use of simple indices of IR in a study are summarised in Table 1. Whilst these are attractive in terms of their ease of use and inexpensive nature, investigators need to be aware of their limitations.

**Table 1:** Criteria for studies in which simple indices of insulin resistance may be used.

	Study objectives
1.	Large clinical practice and epidemiological investigations [70].
2.	Where the assessment of direct IR is not required.
3.	Where the outcome of IR is of secondary interest [71].
4.	Where the requirements of reference techniques are not available *e.g *equipments, trained staff, enough budget.
5.	Where a new simple index is under evaluation [72,73].
6.	Investigating validity and pitfalls of simple indices in specific clinical conditions [41,57].

References have been provided as a study example:

### 1. Oral glucose tolerance test (GTT)-derived indices

Mathematical formulae have been derived which represent the kinetics of insulin and glucose levels at different time intervals before and after administration of a 75 g oral glucose load during an oral glucose tolerance test (GTT). Several IR indices have been derived from two (0 and 120 min) to four (0, 30, 60 and 120 minutes) samples of insulin and glucose taken in the context of a GTT **(**Table 2**)**. More GTT-derived indices have been described which are summarised by Monzillo *et al.*, [7] and Matsuda *et al.*, [21]. Most but not all studies indicate that these indices are more reliable than other simple fasting indices of IR as their correlation with reference techniques was stronger than that of fasting indices [4,9,21-24]. This is to be expected as GTT-derived indices take post-load glucose-insulin interaction into account. Whenever the dynamic techniques cannot be utilised in a study, simple GTT-derived indices should therefore be next in line for quantitation of IR.

**Table 2:** Formulae for the most commonly used simple indices of insulin resistance derived from fasting specimens and other specimens taken in the context of an oral glucose tolerance test (GTT).

Index	Formula	Reference
*GTT derived indices:*		
ISI-gly	2/[(INS_p _× GLY_p_)] + 1	[74]
ISI (composite)	1000/√{[fasting glucose (mg/dL) × fasting insulin (μU/mL)] × [mean glucose × mean insulin]}	[21]
*Fasting sample indices:*		
HOMA-IR	Fasting glucose (mmol/L) ×fasting insulin (mU/L)/22.5	[36]
HOMA2-S	HOMA2 calculator version 2.2	[37]
FGIR	Fasting glucose (mg/dL)/Fasting insulin (mU/L)	[58]
Raynaud	40/Fasting insulin (mU/L)	[33]
Reciprocal insulin	1/fasting insulin (mU/L)	[34]
QUICKI	1/[log fasting insulin (mU/L) + log fasting glucose (mg/dL)]	[46]
FGIR	Fasting glucose (mg/dL)/Fasting insulin (mU/L)	[58]

Key: GTT, oral glucose tolerance test; HOMA-IR, homeostasis model assessment insulin resistance; QUICKI, quantitative insulin sensitivity check index; FGIR, fasting glucose insulin ratio; ISI (composite), an index of whole-body insulin sensitivity; ISI-gly, insulin sensitivity glycemic index; INSp, insulinemic area; GLYp, glycemic area.

#### Physiological response

A criticism of the gold standard test is that the indices derived are not measured under physiological circumstances. When the investigator wishes to use an index that reflects the physiological insulin response to a glucose load then it is appropriate to use one derived from parameters measured during a GTT. These surrogate indices are derived from a dynamic response which generally incorporates both peripheral and hepatic IR. An advantage of the GTT-derived indices over those derived from fasting insulin and glucose alone is that they can detect subtle disturbances in glucose metabolism not apparent from the latter [25,26]. Simple indices based solely on fasting measurements cannot always reliably estimate IR, since it is possible for subjects to be significantly insulin resistant without having fasting hyperinsulinaemia. Furthermore, some individuals may be euglycaemic when fasting but hyperglycaemic and hyperinsulinaemic two hours following a 75 g oral glucose load. It is recognised that even in healthy individuals with hepatic IR, regular diet, exercise or glucose lowering medications can restore both fasting glucose and insulin levels to well within normal ranges [27,28]. In the authors' experience, the larger the number of specimens collected from an individual, the more accurately the derived parameters are likely to reflect insulin sensitivity [29]. This is because the biological variation of insulin and glucose levels has less impact on the value of the parameter when it is derived from a larger quantity of data.

It is noteworthy that the early glucose response during a GTT can be considered an index of hepatic IR, while the drop in glucose levels from peak to nadir estimates peripheral IR predominantly of skeletal muscle with a smaller contribution from adipose tissue [30]. A potential limitation of the GTT is poor reproducibility [31]. This arises from intra-individual variation in glucose handling both pre- and post-absorption. This problem can be reduced by repetition of the test on two or three occasions at short intervals and taking the mean of the results, if the investigator considers this feasible.

### 2. Fasting sample-derived indices

IR indices can be derived from fasting samples using mathematical formulae representing the kinetics of fasting insulin with or without glucose measurement **(**Table 2**)**. These are the most commonly used indices of IR. The reasons for this wide application are their simplicity, cost-effectiveness and practicality as only one fasting sample is required and there is no requirement for administration of a glucose load. The main problems for the investigator are understanding the information that each index provides and choosing one from the large number available. The investigator should be mindful of the fact that these indices vary widely in their reliability [32]. Not all have been rigorously validated against the gold standard. The following points therefore need to be considered before selecting any fasting sample-derived index.

#### Parameters involved in the formula

Many of the formulae for indices derived from fasting specimens are simple using the fasting insulin level alone. An example of this is the Raynaud index which describes the best-fit relationship between fasting insulin and Si [33]. Another simple index is the reciprocal of the fasting insulin level (1/insulin) [34]. In spite of the modification of the original data by such formulae, these indices still rely solely on the insulin level and consequently suffer from the same limitations as the fasting insulin level alone [2]. Formulae which incorporate at least the two main parameters *viz *insulin and glucose, are preferable over those utilising insulin alone. Such formulae represent the exchangeable kinetics between both parameters which ultimately estimate IR. Other formulae have been reported which incorporate more than two parameters for example the lipid-parameter based formula [35]:

Lipid-basedindex=12×[2.5×(HDL-C∕Totalcholesterol)-NEFA]-Fastinginsulin

(HDL-C, high density lipoprotein cholesterol; NEFA, non-esterified fatty acids)

Indices which incorporate more than one parameter have high validity in predicting IR as demonstrated by their close association with reference test-derived indices of IR.

#### Upgraded and modified formulae

Simple IR indices have undergone regular modification by researchers in an effort to improve their applicability. Upgraded formulae have been observed to be more closely associated with reference test-derived indices and consequently more reliable and accurate. For example, the original HOMA equation (HOMA-IR) has been modified to HOMA2 to allow for increased plasma insulin and glucose levels [36,37].

Another means by which equations have been upgraded is incorporating additional parameters into their formulae. For example the fasting non-esterified fatty acid (NEFA) concentration when incorporated into the regular formula of QUICKI (quantitative insulin sensitivity check index) improves the estimation of IR as the modification enhances its correlation with the clamp-based index of insulin sensitivity and its discriminatory power [38,39]. Another example is the use of fasting C-peptide as a substitute for insulin in determining HOMA-IR which can be applied to subjects with insulin-treated diabetes [40]. Researchers using a simple index of IR are advised to use the most up-to-date version in order to obtain the strongest correlation with reference methods.

#### Mathematical considerations

Due to the simple mathematical nature of some fasting sample-derived indices, there is the potential for their output to be misinterpreted in some subjects particularly in those with type 2 diabetes where their levels may be paradoxically and erroneously increased *e.g*. fasting glucose to insulin ratio (FGIR), 1/fasting insulin. This is due to low insulin secretion by pancreatic β-cells [41].

One feature of both the simple and reference method-derived indices of IR is that their values tend to be positively skewed yielding a hyperbolic curve when non-transformed data is examined. They become normally distributed following log-transformation. Previous studies have shown an improvement in their correlation with the reference techniques after log-transformation [4,42-45]. The QUICKI index for example is similar to HOMA, except that it interprets the data by taking both logarithms and the reciprocal of the fasting glucose-insulin product. The log-transformation included in the formula of QUICKI results in greater accuracy than HOMA in calculations over a broad range of insulin sensitivity and in stronger correlation with the HEC (r = 0.78, p < 2 × 10^-12^) [46,47]. Similarly, log or ln transformation of all simple indices of IR results in a stronger association with reference techniques and consequently an improvement in IR estimation. However, the degree of improvement reported varies between indices [4,46].

#### Biological variation

Recent studies have indicated that the biological variation of insulin levels and fasting sample-derived indices of IR are greatly influenced by the degree of glucose tolerance and also by IR-modifying medications [48-50]. Owing to biological variation in fasting insulin levels, values for fasting sample-derived indices are more reliable in subjects with normal glucose tolerance than in individuals with type 2 diabetes. These factors should be borne in mind by researchers carrying out studies which involve repetition of levels at various time intervals. It may be advisable to confirm the glycaemic status by means of a GTT particularly if a long time interval is involved such as during a prospective study, as the glycaemic status may change over the course of the study. Researchers should also be aware that the values for indices which utilise multiple parameters will reflect the biological and analytical variation of each individual parameter. The result obtained for any index is only as good as the data entered.

#### Multiple simple indices

Where there is uncertainty as to which simple index is most suitable in a study, more than one can be used simultaneously *e.g*. HOMA-IR, QUICKI, *etc*. This may help to minimise the effect of limitations of some indices and increase the likelihood of obtaining meaningful differences between categories of subjects under investigation [26,42,51]. In the authors' experience, the results of different simple indices are not always in line with each other. It is therefore wise to use more than one index in a given study in order to avoid reaching erroneous conclusions.

#### Insulin assay

The nature of the insulin assay to be used must be taken into consideration before commencing any study of IR, not only for those using simple indices. Currently all insulin assays are standardised using the same reference preparation but values obtained using different insulin assay kits may show significant bias. This may be due to variable specificity, different calibration set-up in kits or different factors used to convert between units (from mIU/L to pmol/L) which vary from 6.0 to 7.46 [52]. Insulin represents the main parameter involved in the estimation of any simple index estimation. Manley *et al.*, found that the distribution of HOMA2-IR estimates for different insulin assay (11 assays) varied by up to twofold, depending on which insulin assay was used [53]. Bias in results obtained by different insulin assays may contribute to the differences in reported cut-off values for IR in different populations [54,55]. In view of the above, it is highly advisable that, in any given study, one source of insulin assay is used throughout which has high performance criteria (*i.e*. company, antibodies). In addition, the same lot number of the assay products should be used if possible. This is particularly important in large prospective studies.

#### Limitations of simple indices

Indices derived from fasting samples can be unreliable when applied to certain groups of subjects *viz *the elderly and those with uncontrolled diabetes or type 1 diabetes [46,56,57]. The measured insulin level will not accurately reflect the degree of IR in these individuals as the β-cells are unable to secrete sufficient insulin to overcome existing IR. Indices derived from fasting samples are therefore more reliable when applied to individuals with sufficient insulin secretion. For example FGIR has been observed to be reliable in subjects with polycystic ovary syndrome (PCOS) except those who also have type 2 diabetes [41,58]. As is the case with the reference techniques, it is therefore advisable, when using simple indices, that the subject's glycaemic status is classified beforehand by means of a GTT and WHO criteria, even where post-load values are not required for determination of the index itself.

Simple indices based on fasting levels of glucose and insulin (*e.g*. HOMA-IR and QUICKI) assess hepatic IR more than peripheral insulin sensitivity. This has been demonstrated by the strong relationship observed between simple indices and the reference parameter of hepatic IR obtained by Hoffman *et al*., utilising FSIVGTT technique [59]. Hepatic IR is considered the major determinant of fasting hyperglycaemia and as such is the major factor contributing to the pre-diabetic state, impaired fasting glucose [18,19]. In most circumstances peripheral tissue IR develops later than hepatic IR [19]. This is an important limitation of fasting simple indices. Whilst hepatic and peripheral IR correlate with each other the relative contribution of each varies between individuals.

### 3. Biochemical markers of IR

The concentration of a given protein obtained from a fasting blood sample is potentially a more convenient means of assessing IR than measuring glucose and insulin together. Many possible biochemical markers were discussed in our previous review [2].

#### Degree of insulin resistance

Other than the fasting insulin level itself, initial studies have indicated that proteins which are either directly or indirectly insulin-regulated are more reliable as markers of IR than insulin independent protein markers [3,5,60,61]. In individuals with normal glucose tolerance the application of these biochemical markers is very reliable and convenient as they have been observed to be highly associated with IR parameters determined by reference techniques. However, they have been noted to be less-reliable in subjects with more marked degrees of IR resulting in an abnormal glucose tolerance [4,5,62]. This observation may be due to the presence of hepatic IR and consequently irregular secretion of these markers by hepatocytes as liver is considered to be the main source of most of these biochemical markers.

#### Ratio between different markers

The ratio of biochemical markers other than insulin and glucose may be another option for a simple and efficient marker of IR. For example, the ratios of leptin to adiponectin or between triglycerides and high density lipoprotein cholesterol can be considered useful markers of IR [63-65]. Low triglyceride levels are usually associated with increased insulin sensitivity but it appears that the relationship between triglycerides and insulin sensitivity differs between races, African-Americans for example having lower triglyceride levels in spite of increased insulin resistance [66]. Therefore, as with all biochemical markers, the results of these indices should not be interpreted in isolation but in the context of other findings including anthropometric measurements such as body mass index (BMI) and waist circumference and biochemical measurements. Age, gender, race *etc*. should be considered also.

#### Biological variation for different markers

The issue of biological variation as discussed above in relation to fasting indices, also applies to single biochemical markers of IR. Jaygobal *et al.*, previously investigated the biological variation of sex hormone binding globulin (SHBG) as a biochemical marker of IR [67] and found that a second level must rise or fall by > 14.5% to be considered significantly different from the first in subjects with type 2 diabetes. Similarly, we assessed the biological variation of serum IGFBP-1 in individuals with different degrees of glucose tolerance [68]. Our study showed that biological variation of IGFBP-1 is lowest in normal glucose tolerant individuals and increased with deteriorating glucose tolerance. Therefore, biological variation should be considered whenever biochemical markers, influenced directly or indirectly by insulin, are utilised to assess IR. Generally, biological variation for most of the analytes and proteins including other biochemical markers can be retrieved from the database using the following website (http://www.westgard.com/biodatabase1.htm) [69].

## Conclusions

A wide variety of methods are available for assessing IR including reference techniques and simple indices. In planning studies on IR and selecting a suitable index, a number of important factors need to be considered by investigators, the principle one being the nature of the study to be undertaken. Where possible the HEC, as the most accurate technique available, remains the first choice but simpler and inexpensive methods may be appropriate provided the investigator is aware of their limitations.

## List of abbreviations

AIR: acute insulin response; AUC: area under the curve; CIGMA: continuous infusion of glucose with model assessment; DI: disposition index; FSIVGTT: frequently sampled intravenous glucose tolerance; FGIR: fasting glucose insulin ratio; GTT: glucose tolerance test; HEC: hyperinsulinaemic euglycaemic clamp; HOMA: homeostasis model assessment; HGP: hepatic glucose production; IR: insulin resistance; ISI-(gly): insulin sensitivity glycaemia index; ITT: insulin tolerance test; Sg: glucose effectiveness; NEFA: fasting non-esterified fatty acid; QUICKI: quantitative insulin sensitivity check index; %β-cell: β-cell activity

## Competing interests

The authors declare that they have no competing interests.

## Authors' contributions

The work presented here was carried out in collaboration between all authors. All authors read and approved the final manuscript. The individual contributions of authors to the manuscript are as follow - AB: the main author who wrote the paper, designed the main theme, titles and subtitles. It was his idea to write the article. CL: co-designer for the main titles and subtitles, filled the gaps between lines and main manuscript, English Language Check. IK: the article's main reviewer, provided additional references and comments. GF: final revision before submission, added many comments and suggestions, English Language Check.

## Pre-publication history

The pre-publication history for this paper can be accessed here:

http://www.biomedcentral.com/1471-2288/11/158/prepub
